# Curcumin Nicotinate Selectively Induces Cancer Cell Apoptosis and Cycle Arrest through a P53-Mediated Mechanism

**DOI:** 10.3390/molecules24224179

**Published:** 2019-11-18

**Authors:** Ying-chun He, Lan He, Ramina Khoshaba, Fang-guo Lu, Chuan Cai, Fang-liang Zhou, Duan-fang Liao, Deliang Cao

**Affiliations:** 1 Hunan Provincial Key Laboratory for Prevention and Treatment of Ophthalmology and Otolaryngology Diseases with Chinese Medicine, State Key Laboratory of Chinese Medicine Powder and Medicine Innovation in Hunan (incubation), College of Medicine, Hunan University of Chinese Medicine, Changsha 410208, Chinaisland365@163.com (L.H.); chuancai198910@gmail.com (C.C.);; 2 Department of Medical Microbiology, Immunology & Cell Biology, Simmons Cancer Institute, Southern Illinois University School of Medicine. 913 N. Rutledge Street, Springfield, IL 62794, USA; ramina_1981@yahoo.com; 3 Department of Biotechnology, College of Science, University of Baghdad, Baghdad 10011, Iraq

**Keywords:** curcumin, curcumin nicotinate, p53, apoptosis, cell cycle arrest

## Abstract

Curcumin is an anticancer agent, but adverse effects and low bioavailability are its main drawbacks, which drives efforts in chemical modifications of curcumin. This study evaluated antiproliferative activity and cancer cell selectivity of a curcumin derivative, curcumin nicotinate (CN), in which two niacin molecules were introduced. Our data showed that CN effectively inhibited proliferation and clonogenic growth of colon (HCT116), breast (MCF-7) and nasopharyngeal (CNE2, 5-8F and 6-10B) cancer cells with IC_50_ at 27.7 μM, 73.4 μM, 64.7 μM, 46.3 μM, and 31.2 μM, respectively. In cancer cells, CN induced apoptosis and cell cycle arrest at G2/M phase through a p53-mediated mechanism, where p53 was activated, p21 and pro-apoptotic proteins Bid and Bak were upregulated, and PARP was cleaved. In non-transformed human mammary epithelial cells MCF10A, CN at 50 µM had no cytotoxicity and p53 was not activated, but curcumin at 12.5 µM activated p53 and p21 and inhibited MCF10A cell growth. These data suggest that CN inhibits cell growth and proliferation through p53-mediated apoptosis and cell cycle arrest with cancer cell selectivity.

## 1. Introduction

**Figure 1:**
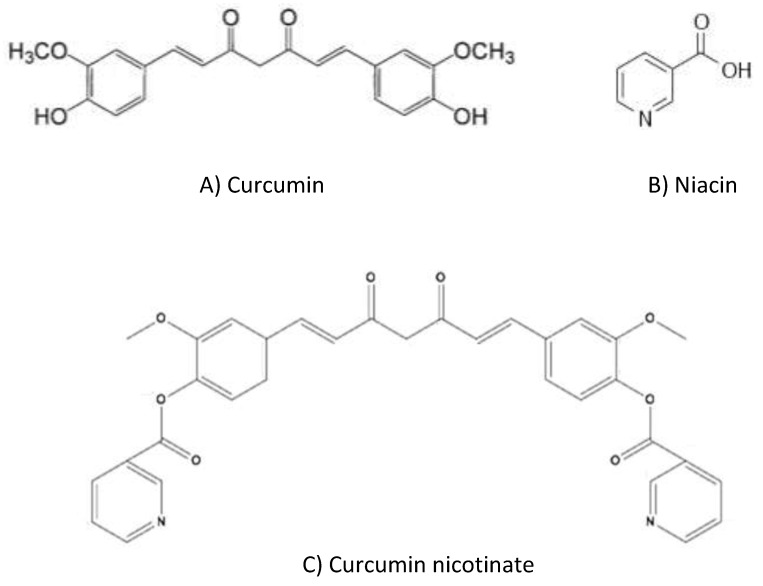
Chemical formulas of curcumin, niacin and curcumin nicotinate.

Cancers are the most common and deadliest diseases threatening human lives around the world; a worse scenario is the increasing morbidity and mortality of cancer annually. Chemotherapy is an indispensable option thus far in the treatment of metastatic or relapsed cancer [1]. Curcumin (diferuloylmethane, Figure 1A) is a phenolic color pigment, widely existing in rhizome of plants, such as *Curcuma longa*, *curcuma, zedoary, Acorus calamus* and *zedoary turmeric*. Curcumin has shown a variety of pharmacological effects, such as anti-inflammatory, anti-oxidant, anti-angiogenesis, analgesic and antiseptic activity [2,3]. Curcumin also demonstrates potent antiproliferative activity in a wide range of cancer cells, including liver, breast, lung, stomach, colon, prostate, head and neck cancers [4,5,6,7,8,9,10]. Curcumin exerts anti-tumor activity through multiple mechanisms, such as apoptosis [11], cell cycle arrest [12] and anti-angiogenesis [13]. Curcumin also enhances cell sensitivity to chemotherapy drugs by suppression of multidrug resistant gene expression [14,15].

Curcumin causes side effects. Participants may experience diarrhea and nausea when curcumin is supplemented at 0.45 to 3.6 g/day in clinical trials [16]. Serum alkaline phosphatase and lactate dehydrogenase increased in some participants with curcumin supplements [17]. Single oral dosages of curcumin at 12 g resulted in diarrhea, headache, rash and yellow stool [18]. In a phase II trial, abdominal pain was reported in cases with curcumin treatment at 8 g/day [19]. Curcumin also has defects in clinic application, such as poor water solubility, instability and low oral bioavailability [20,21,22]. Curcumin levels in serum were peaked at 0.41 to 1.75 μM one hour after oral administration of 4 to 8 g of curcumin [23], and concentrations in plasma was approximately 0.01 μM only after oral administration of 3.6 g curcumin [16].

Niacin (also known as nicotinic acid or vitamin B_3_) is a colorless, water-soluble organic compound with a carboxyl group (COOH) at the 3-position of pyridine (Figure 1B). Niacin and its derivative nicotinamides, nicotinamide adenine dinucleotide (NAD) and nicotinamide adenine dinucleotide phosphate (NADP), are important coenzymes in live cells, participating in various hydrogen transfer processes [24]. Therefore, organs with a high energy need (e.g., brain) or a high cell turnover rate (e.g., gastrointestinal epithelium) are highly susceptible to niacin deficiency that causes nausea, mouth lesions, anemia, headache and tiredness [24,25]. In addition, niacin is also involved in cell signaling transduction, DNA repair and steroid hormone production [26,27]. Therefore, niacin is an important protective vitamin in cells.

The combination of agents with different biological effects is a well-practiced philosophy in chemotherapy of cancer [28,29]. Taking the anti-cancer activity of curcumin and metabolic importance of niacin, an inter-molecule combination of these two agents was designed to produce a new compound, named curcumin nicotinate (CN) (Figure 1C), where two niacin molecules were introduced into a curcumin and improved the solubility and stability [30,31]. This study evaluated the antiproliferative activity and cancer cell selectivity of CN.

## 2. Results

### 2.1. Curcumin Nicotinate Has Antiproliferative Activity in Multiple Cancer Cell Lines

**Figure 2:**
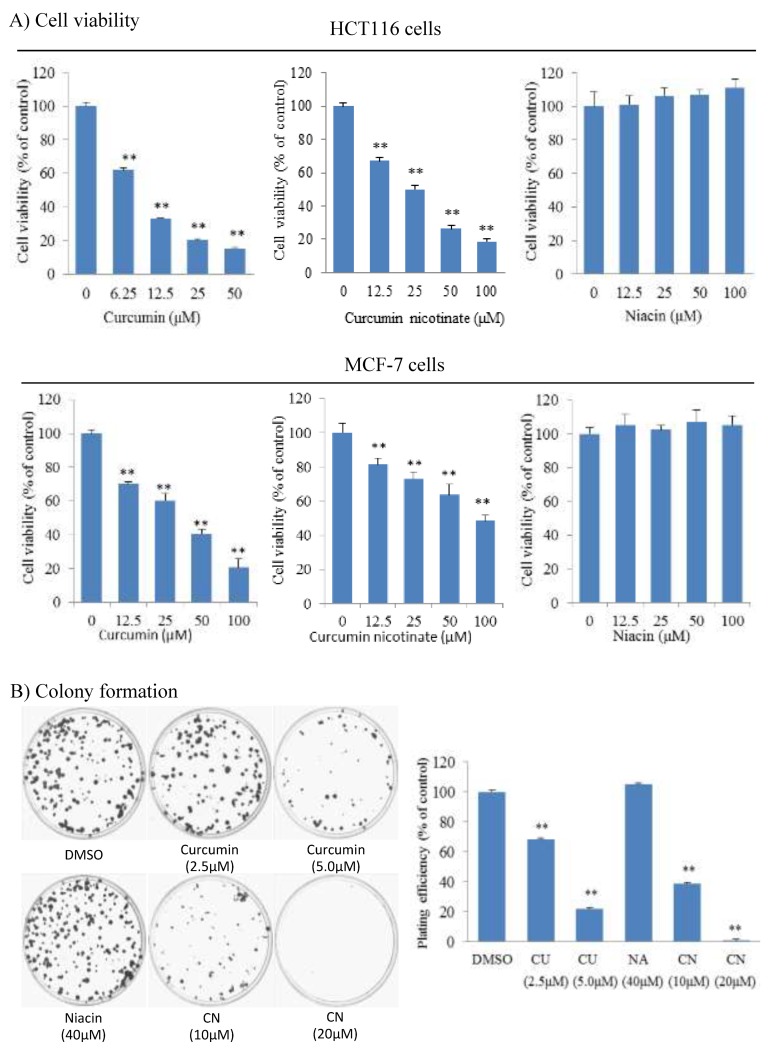
Anti-proliferative activity of curcumin nicotinate. (**A**) Cell viability. HCT116 and MCF-7 cells were exposed to niacin, curcumin nicotinate or curcumin at concentrations indicated for 72 h. The percent of viable cells were determined by MTT assays as described in Materials and Methods. (**B**) Colony formation assay. HCT116 cells were seeded in 6cm plates for 24 hours, followed by exposure for 14 days to mock (1% DMSO), niacin, curcumin nicotinate or curcumin. After being stained with crystal violet for 10 min, colonies were photographed and colony formation efficiency was calculated as described in Materials and Methods. Right panel: Plating efficiency normalized to mock control group (DMSO). Data denote the mean ± SD from three independent experiments. Data were analyzed by one-way ANOVA analysis. ** *p* < 0.01 compared to mock control cells. NA, niacin; CN, curcumin nicotinate; and CU, curcumin.

We tested antiproliferative activity of curcumin nicotinate (CN) in multiple cell lines, including human colon (HCT116), breast (MCF-7) and nasopharyngeal (CNE2, 5-8F and 6-10B) cancer cell lines. Results showed that CN demonstrated antiproliferative activity in all these tested cell lines (Figure 2A and Figure S1). The IC_50_ of CN was at 27.7 μM in HCT116 cells, 73.4 μM in MCF-7 cells, 64.7 μM in CNE2 cells, 46.3 μM in 5-8F cells and 31.2 μM in 6-10B cells. It appeared that HCT116 and 6-10B cells were more sensitive than MCF-7, CNE2, and 5-8F (*p* < 0.05). HCT116 was derived from colon cancer and MCF-7 was established from breast cancer. Colon and breast cancers are the most popular tumors in Western populations, and thus further studies were focused on these two cell lines. As documented in literature [32], curcumin inhibited cell growth and proliferation, but niacin did not (Figure 2A and Figure S1). We further assessed the activity of CN in inhibition of clonogenic growth of cancer cells. As shown in Figure 2B, CN effectively inhibited the colony-formation and growth of cancer cells. Colony forming rates were at 38.6% and 0.8% in presence of CN at 10 μM and 20 μM, respectively. Together these data indicate that CN has antiproliferative activity. 

### 2.2. Curcumin Nicotinate Induces Apoptosis and Cell Cycle Arrest 

**Figure 3:**
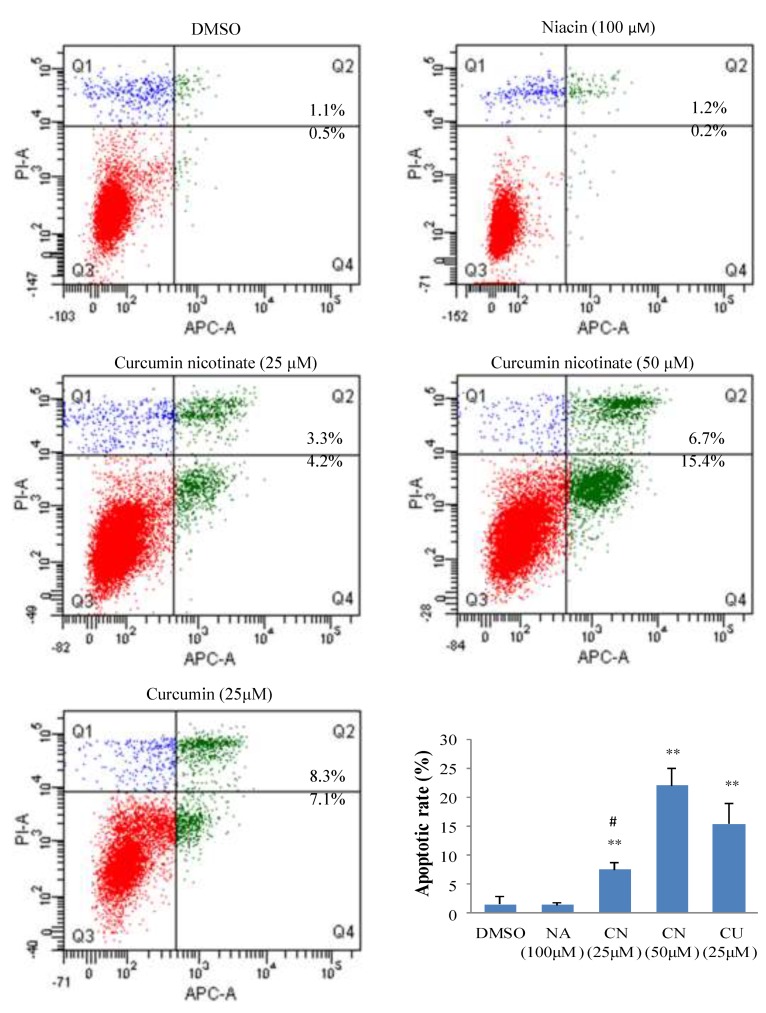
Apoptosis induced by curcumin and curcumin nicotinate. HCT116 cells were treated for 36 h with mock (DMSO), niacin, curcumin nicotinate or curcumin and then collected for apoptosis by flow cytometry as described in Materials and Methods. Q2 phase indicates late apoptosis and Q4 phase denotes early apoptosis. Apoptotic rate was calculated as the total cells in Q2 and Q4 phases. Data represent the mean ± SD from three independent experiments. Data were analyzed by one-way ANOVA analysis. ** *p* < 0.01 compared to control cells; # *p* < 0.05 compared to CU at 25 µM. NA, niacin; CN, curcumin nicotinate; and CU, curcumin.

**Figure 4:**
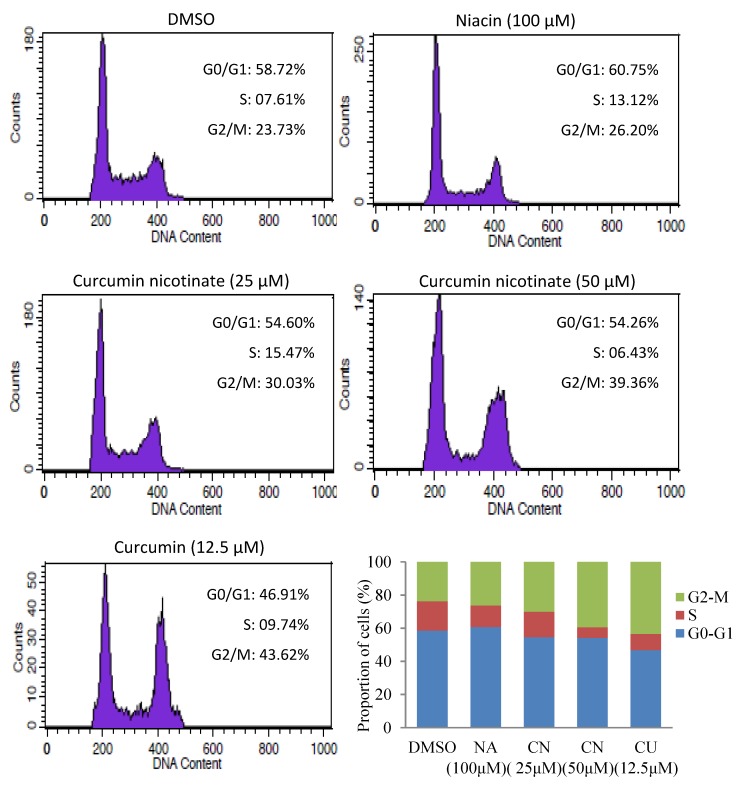
Cell cycle arrest induced by curcumin nicotinate. HCT116 cells were treated for 36 h with mock (DMSO), niacin, curcumin nicotinate or curcumin and then collected for cell cycle distribution analysis as described in Materials and Methods. NA, niacin; CN, curcumin nicotinate; and CU, curcumin.

To understand the underlying mechanisms of antiproliferative activity of CN, we assessed apoptosis in CN-treated cells. As shown in Figure 3, CN at 25 μM triggered cancer cell apoptosis, and vast apoptosis occurred when the CN was increased to 50 μM. CN-induced apoptosis in cancer cells was further confirmed by AO/EB staining (Figure S2). Like reports in literature [33,34], curcumin also induced apoptosis, but niacin did not (Figure 3). We further evaluated cell cycle distribution in cancer cells treated by CN. The results showed that like curcumin, CN induced cell cycle arrest at G2/M phase, but niacin did not (Figure 4 and Figure S3). 

### 2.3. Curcumin Nicotinate Induces Cell Cycle Arrest and Apoptosis Through a p53-Mediated Mechanism

**Figure 5:**
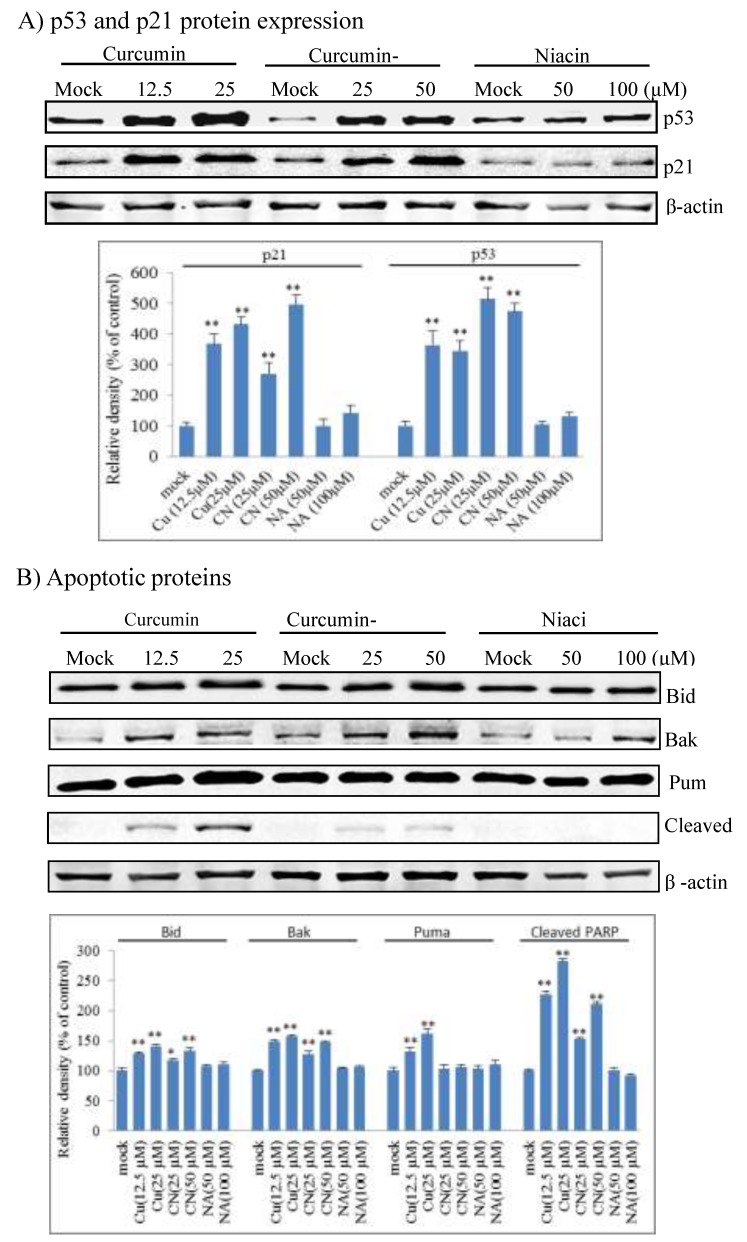
Induction of cell cycle inhibition and apoptotic proteins by curcumin nicotinate. HCT116 cells were treated for 36 h with mock (DMSO), niacin, curcumin nicotinate or curcumin and then collected for Western blot analysis as described in Materials and Methods. Housekeeping β-actin served as a loading control. (**A**) P53 and p21 proteins. (**B**) Apoptotic proteins. Representative images are exhibited; values indicate the mean ± SD of three repeats. * *p* < 0.05 and ** *p* < 0.01 compared to mock control cells. NA, niacin; CN, curcumin nicotinate; and CU, curcumin.

We further explored effector proteins that triggered cell cycle arrest and apoptosis in CN-treated cancer cells. As show in Figure 5A, CN activated p53 and induced p21 expression in a dose-dependent manner. P21 is a major cell cycle inhibitor [35] and thus triggered the cell cycle arrest in response to CN. We further evaluated p53-targeted apoptotic proteins in CN-treated cells, and results showed that CN triggered apoptotic Bid and Bak protein expression and PARP cleavage, but did not alter Puma expression (Figure 5B). Curcumin also triggered p53 activation and p21 and apoptotic Bid and Bak protein induction, but niacin did not. Together these data suggest that CN induces apoptosis and cell cycle arrest through a p53-mediated mechanism. 

### 2.4. Curcumin Nicotinate Demonstrates Minimal Antiproliferative Activity in Non-Transformed MCF10A Cells

**Figure 6:**
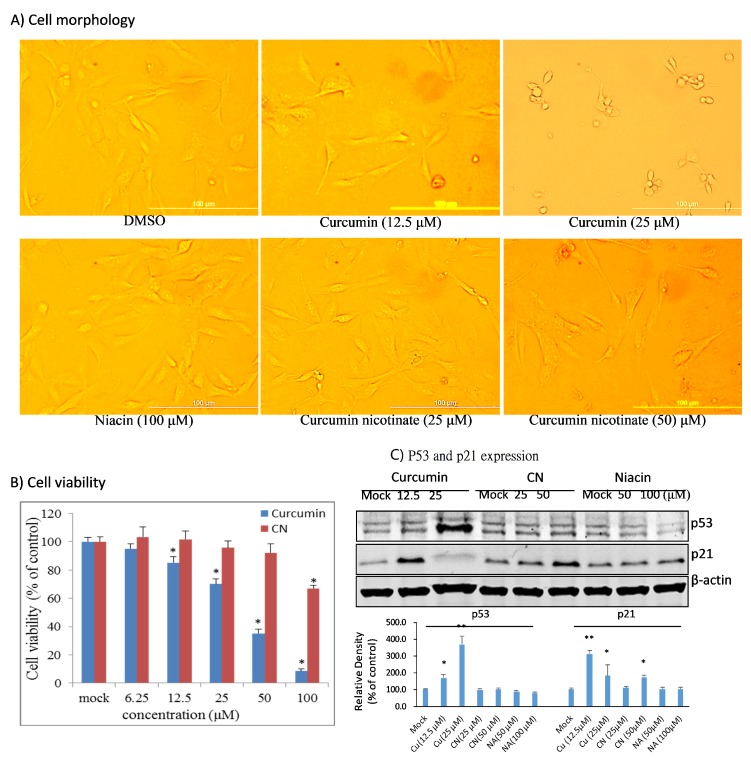
Minimized anti-proliferative activity of curcumin nicotinate in MCF10A cells. (**A**) Cell morphology. MCF10A cells were treated for 36 h with mock (DMSO), niacin, curcumin nicotinate or curcumin and subjected to photographing under an inverted microscopy. (**B**) Cell viability. MCF10A cells were treated for 72 h with mock, curcumin nicotinate or curcumin. Viable cells were determined by MTT assays as described in Materials and Methods. Data represent the mean ± SD from three independent experiments. Data were analyzed by one-way ANOVA analysis. * *p* < 0.05 compared to mock control cells. (**C**) P53 and p21 expression. MCF10A cells were treated for 36 h with mock (DMSO), niacin, curcumin nicotinate or curcumin and then collected for Western blot analysis as described in Materials and Methods.

Non-selective cytotoxicity is the main drawback of chemotherapeutic agents; tremendous efforts have been invested thus far to improve the cancer cell selectivity of chemotherapy. After we confirmed the antiproliferative activity of CN, we evaluated its cytotoxicity in non-transformed human mammary epithelial cells MCF10A in parallel with curcumin. As shown in Figure 6A and B, CN had no noticeable cytotoxicity in MCF10A at a concentration of 50 μM that triggered vast apoptosis and cell cycle arrest in multiple cancer cell lines. In sharp contrast, curcumin at 12.5 μM demonstrated significant cytotoxicity in MCF10A cells, and at a concentration of 50 μM, curcumin killed approximately 70% of MCF10A cells (Figure 6B). Further studies showed that CN at 50 μM had no effects on p53 and p21 expression, but curcumin at 12.5 μM did (Figure 6C). Together these data suggest that CN has better cancer cell selectivity than curcumin.

## 3. Discussion

Curcumin is a natural yellow small polyphenol compound extracted from the rhizome of the plants [18]. Curcumin is considered as an active anticancer agent in a wide range of tumors. Through induction of apoptosis and cell cycle arrest, curcumin inhibits tumor growth and progression [36,37]. However, the clinical application of curcumin has been limited because of low stability, bioavailability and selectivity [20,38]. Chemical modifications and novel pharmacological delivery methods of curcumin, including new derivatives and nanocarriers, such as conjugates, nanoparticles, micelles, solid dispersions and liposomes, have been developed to solve these shortcomings and improve the clinical applications [39,40,41]. These efforts improved bioavailability of curcumin, but not the selectivity. Curcumin nicotinate (CN) is a new derivative synthesized by introducing two niacin molecules into curcumin. The hypothetic strategy of this modification is to take advantage of niacin as an important nutrient needed by normal cells for proper function [25]. Niacin is a water-soluble vitamin B3, and as a major component of coenzymes NAD and NADP, niacin participates in a variety of cellular activities, including energy metabolism, signaling transduction and DNA synthesis and repair [24]. Delivery of niacin with curcumin as a single molecule may improve the cancer cell selectivity of curcumin through reducing cytotoxicity to normal cells.

To test this hypothesis, we first proved the antiproliferative activity of CN in multiple cancer cells, including HCT116, MCF-7, CNE2, 5-8F and 6-10B cells. CN also inhibited clonogenic growth of tested cancer cells, indicating its potential as anticancer agent. Subsequent studies revealed that CN induced apoptosis and cell cycle arrest at the G2/M phase through p53-mediated mechanisms, confirming its antiproliferative activity. Therefore, similar to its parental curcumin, CN holds the antiproliferative activity, functioning through the p53-mediated apoptosis and cell cycle arrest, but has lower antiproliferative activity. This may be ascribed to the metabolic protection of niacin introduced into the molecule CN. Further study is warranted to address this question.

Impressively, this niacin modified curcumin derivative CN demonstrated improved cancer cell selectivity. At 50 μM, a concentration that induced apoptosis and cell cycle arrest in cancer cell lines, CN had not measurable cytotoxicity in the non-transformed MCF10A cells. Neither p53 activation nor p21 induction was detected in CN-treated MCF10A cells, but in contrast, curcumin at 12.5 μM activated p53 and p21 and inhibited growth and proliferation of MCF10A cells, suggesting the selective cytotoxicity of CN in cancer cells. This may be ascribed to protective role of niacin, an important nutrient in cellular metabolism. 

## 4. Materials and Methods

### 4.1. Antibody, Drugs and Chemicals

Mouse monoclonal anti-β-actin, rabbit polyclonal anti-bcl-2, rabbit polyclonal anti-Puma, rabbit polyclonal anti-cleaved-Bid, rabbit polyclonal anti-Bak, rabbit polyclonal anti-cleaved PARP, rabbit polyclonal anti-p53 and rabbit polyclonal anti-P21 antibodies were purchased from Cell Signaling Technology (Danvers, MA, USA). Horseradish peroxidase linked secondary sheep anti-mouse and rabbit anti-horse IgG were obtained from GE Healthcare (Chicago, IL, USA). Propidium iodide (PI) and Annexin V were purchased from BD Pharmingen (Franklin Lakes, NJ, USA). Complete lysis-M was purchased from Roche Applied Science (Indianapolis, IN, USA). AO/EB nuclear dyes were purchased from Beyotime (Shanghai, China). Curcumin, niacin, 3-[4–dimethylthiazol-2-yl]-2, 5-diphenyltetrazoliumbromide (MTT) and other chemicals were purchased from Sigma-Aldrich (St Louis, MO, USA). Curcumin nicotinate (CN) was in-home synthesized. For experimental uses, CN was dissolved in DMSO at 10 mM as stock solution and then diluted to concentrations indicated.

### 4.2. Cell Lines and Culture Conditions

Cell lines HCT116, MCF-7 and MCF10A were purchased from American Type Culture Collection (Manassas, VA, USA); 5-8F, 6-10B and CNE2 cell lines were purchased from Zhongshan University, Guangzhou, China. Cells were maintained in RPMI 1640 or DMEM with 10% fetal bovine serum and penicillin/streptomycin (100 U/mL and 100 mg/mL) at 37 °C in 5% CO_2_.

### 4.3. Cell Viability Assay

Cell viability was quantitated by MTT. Cells (4–5 × 10^3^ cells/well) were seeded in 96-well plates and incubated overnight. Next day, cells were fed with fresh medium containing niacin, curcumin or curcumin nicotinate at indicated concentrations with 8 duplicated wells at each concentration. After 72 hours, MTT was added to each well and incubated at 37 °C for 4 h. Formazan products of MTT were dissolved in DMSO and measured as absorbance at 570 nm in a spectrophotometer (Thermo Fisher, FL, USA). Percentage of viable cells was expressed as ratio over the control [42].

### 4.4. Colony Formation Assay

Cells (500 cells/well) were seeded in 6 cm plates overnight. Niacin, curcumin or curcumin nicotinate was added at indicated concentrations with three replicates for each concentration. Cells were cultured in a humidified 5% CO_2_ atmosphere at 37 °C for 14 days, and medium was changed every 4 days. At the end of the incubation, culture medium was decanted and each plate was washed twice with PBS (phosphate-buffered saline). Cell colonies were stained with crystal violet for 10 min and counted. Plating efficiency (%) was calculated by colony number over cells seeded [43].

### 4.5. Apoptosis Assay

Apoptosis was estimated by flow cytometry and acridine orange/ethidium bromide (AO/EB) staining. For flow cytometry assays, approximately 1 × 10^6^ cells were harvested. After being washed with PBS containing 2% horse serum and then with binding buffer (10 mM Hepes/NaOH, 140 mM NaCl, 2.5 mM CaCl_2_, pH7.4), the cells were re-suspended in 100 µl binding buffer containing Annexin V and PI. After incubation at room temperature for 10–15 min, cells were submitted to a flow cytometer (E5464, Becton Dickinson, Coppell, TX, USA) [44]. For AO/EB staining, cells were washed with PBS three times and stained with 1.0 μg/mL of AO/EB nuclear dye (Beyotime, Shanghai, China) for 5 min at room temperature. Images were taken using fluorescence microscopy (Nikon Ti-S, Tokyo, Japan).

### 4.6. Cell Cycle Analysis

Approximately 1 × 10^6^ cells were harvested, rinsed twice with cold PBS and then suspended in 500 µl PBS, followed by fixation with 1.5 mL 100% ethanol at 4 °C overnight. After washed with PBS, cells were re-suspended in 200 µl PBS with 3.8 mM sodium citrate (pH 7.2), PI (540 µg/mL) and 200 µg RNase at 4 °C overnight. Stained cells were analyzed for DNA contents by a flow cytometer (E5464, Becton Dickinson, Coppell, TX, USA).

### 4.7. Western Blot Analysis

Cells were washed with cold PBS and lysed on ice in complete lysis-M buffer (Roche, IN, USA) containing a protease inhibitor cocktail. Lysates were centrifuged at 20,800 ×g for 10 min at 4 °C, and supernatant was collected. Protein separation, membrane blotting and antibody probing were conducted as previously described [45]. Band density was quantified by a densitometry (LI-COR, Lincoln, NE, USA).

### 4.8. Statistical Analysis

Data were analyzed by IBM SPSS version 17 software (Armonk, NY, USA). Analysis of variance was used for comparison of cell viability, colony-forming efficiency, apoptosis and protein expression levels. *P*-values less than 0.05 were considered statistically significant.

## 5. Conclusions

Curcumin derivative CN demonstrated antiproliferative activity in several cancer cell lines from different types of cancers through triggering p53-mediated apoptosis and cell cycle arrest. Compared to the parental curcumin, CN remains the antiproliferative activity and mechanism of action, but demonstrates cancer cell selectivity, thus being a promising therapeutic agent of cancer.

## Supplementary Materials

The following are available online, Figure S1: Antiproliferative activity of curcumin nicotinate in nasopharyngeal cancer cells, Figure S2: Curcumin nicotinate-induced apoptosis detected by AO/EB staining, Figure S3: MCF-7 cell cycle arrest induced by curcumin nicotinate.

## Abbreviations Used

AO, acridine orange; CN, curcumin nicotinate; CU, curcumin; DMSO, dimethyl sulfoxide; EB, ethidium bromide; MTT, 3-[4,5-dimethylthiazol-2-yl]-2, 5-diphenyltetrazoliumbromide; NA, niacin; NAD, nicotinamide adenine dinucleotide; NADP, nicotinamide adenine dinucleotide phosphate; PBS, phosphate-buffered saline; VEGF, vascular endothelial growth factor.

## References

[1] Luo Q., Zhang L., Luo C., Jiang M. (2019). Emerging strategies in cancer therapy combining chemotherapy with immunotherapy. Cancer Lett..

[2] Egan M.E., Pearson M., Weiner S.A., Rajendran V., Rubin D., Glockner-Pagel J., Canny S., Du K., Lukacs G.L., Caplan M.J. (2004). Curcumin, a major constituent of turmeric, corrects cystic fibrosis defects. Science.

[3] Rajitha B., Belalcazar A., Nagaraju G.P., Shaib W.L., Snyder J.P., Shoji M., Pattnaik S., Alam A., El-Rayes B.F. (2016). Inhibition of NF-kappaB translocation by curcumin analogs induces G0/G1 arrest and downregulates thymidylate synthase in colorectal cancer. Cancer Lett..

[4] Gao W., Chan J.Y., Wei W.I., Wong T.S. (2012). Anti-cancer effects of curcumin on head and neck cancers. Anticancer Agents Med. Chem..

[5] Bandyopadhyay D. (2014). Farmer to pharmacist: Curcumin as an anti-invasive and antimetastatic agent for the treatment of cancer. Front. Chem..

[6] Abouzied M.M., Eltahir H.M., Abdel Aziz M.A., Ahmed N.S., Abd El-Ghany A.A., Abd El-Aziz E.A., Abd El-Aziz H.O. (2015). Curcumin ameliorate DENA-induced HCC via modulating TGF-beta, AKT, and caspase-3 expression in experimental rat model. Tumour Biol..

[7] Fan Z., Duan X., Cai H., Wang L., Li M., Qu J., Li W., Wang Y., Wang J. (2015). Curcumin inhibits the invasion of lung cancer cells by modulating the PKCalpha/Nox-2/ROS/ATF-2/MMP-9 signaling pathway. Oncol. Rep..

[8] Abuelba H., Cotrutz C.E., Stoica B.A., Stoica L., Olinici D., Petreus T. (2015). In vitro evaluation of curcumin effects on breast adenocarcinoma 2D and 3D cell cultures. Rom. J. Morphol. Embryol..

[9] Bimonte S., Barbieri A., Palma G., Rea D., Luciano A., D’Aiuto M., Arra C., Izzo F. (2015). Dissecting the role of curcumin in tumour growth and angiogenesis in mouse model of human breast cancer. BioMed Res. Int..

[10] Lee Y.H., Song N.Y., Suh J., Kim D.H., Kim W., Ann J., Lee J., Baek J.H., Na H.K., Surh Y.J. (2018). Curcumin suppresses oncogenicity of human colon cancer cells by covalently modifying the cysteine 67 residue of SIRT1. Cancer Lett..

[11] Seo J.A., Kim B., Dhanasekaran D.N., Tsang B.K., Song Y.S. (2016). Curcumin induces apoptosis by inhibiting sarco/endoplasmic reticulum Ca^2+^ ATPase activity in ovarian cancer cells. Cancer Lett..

[12] Jiang J., Cao Y., Mo N., Li J., Mo X. (2012). Curcurmin inhibits on proliferation in A549 cells through P53/P21/PCNA/elF4E signaling pathway. J. Third Mil. Med. Univ..

[13] El-Azab M., Hishe H., Moustafa Y., El-Awady el S. (2011). Anti-angiogenic effect of resveratrol or curcumin in Ehrlich ascites carcinoma-bearing mice. Eur. J. Pharm..

[14] Sreenivasan S., Ravichandran S., Vetrivel U., Krishnakumar S. (2013). Modulation of multidrug resistance 1 expression and function in retinoblastoma cells by curcumin. J. Pharm. Pharm..

[15] Lu W.D., Qin Y., Yang C., Li L., Fu Z.X. (2013). Effect of curcumin on human colon cancer multidrug resistance in vitro and in vivo. Clinics.

[16] Sharma R.A., Euden S.A., Platton S.L., Cooke D.N., Shafayat A., Hewitt H.R., Marczylo T.H., Morgan B., Hemingway D., Plummer S.M. (2004). Phase I clinical trial of oral curcumin: Biomarkers of systemic activity and compliance. Clin. Cancer Res..

[17] Sharma R.A., Gescher A.J., Steward W.P. (2005). Curcumin: The story so far. Eur. J. Cancer.

[18] Lao C.D., Ruffin M.T., Normolle D., Heath D.D., Murray S.I., Bailey J.M., Boggs M.E., Crowell J., Rock C.L., Brenner D.E. (2006). Dose escalation of a curcuminoid formulation. BMC Complement. Altern. Med..

[19] Epelbaum R., Schaffer M., Vizel B., Badmaev V., Bar-Sela G. (2010). Curcumin and gemcitabine in patients with advanced pancreatic cancer. Nutr. Cancer.

[20] Anand P., Kunnumakkara A.B., Newman R.A., Aggarwal B.B. (2007). Bioavailability of curcumin: Problems and promises. Mol. Pharm..

[21] Yang K.Y., Lin L.C., Tseng T.Y., Wang S.C., Tsai T.H. (2007). Oral bioavailability of curcumin in rat and the herbal analysis from Curcuma longa by LC-MS/MS. J. Chromatogr. B Anal. Technol. Biomed. Life Sci..

[22] Garcea G., Jones D.J., Singh R., Dennison A.R., Farmer P.B., Sharma R.A., Steward W.P., Gescher A.J., Berry D.P. (2004). Detection of curcumin and its metabolites in hepatic tissue and portal blood of patients following oral administration. Br. J. Cancer.

[23] Cheng A.L., Hsu C.H., Lin J.K., Hsu M.M., Ho Y.F., Shen T.S., Ko J.Y., Lin J.T., Lin B.R., Ming-Shiang W. (2001). Phase I clinical trial of curcumin, a chemopreventive agent, in patients with high-risk or pre-malignant lesions. Anticancer Res..

[24] Kennedy D.O. (2016). B Vitamins and the Brain: Mechanisms, Dose and Efficacy—A Review. Nutrients.

[25] Jacobson M.K., Jacobson E.L., Chang P. (2018). Vitamin B3 in Health and Disease: Toward the Second Century of Discovery. ADP-Ribosylation and NAD+ Utilizing Enzymes.

[26] Kirkland J.B. (2012). Niacin requirements for genomic stability. Mutat. Res..

[27] Surjana D., Halliday G.M., Damian D.L. (2010). Role of nicotinamide in DNA damage, mutagenesis, and DNA repair. J. Nucleic Acids.

[28] Kiesewetter B., Mayerhoefer M.E., Lukas J., Zielinski C.C., Mullauer L., Raderer M. (2014). Rituximab plus bendamustine is active in pretreated patients with extragastric marginal zone B cell lymphoma of the mucosa-associated lymphoid tissue (MALT lymphoma). Ann. Hematol..

[29] El Weshi A., Memon M., Raja M., Bazarbashi S., Rahal M., El Foudeh M., Pai C., Allam A., El Hassan I., Ezzat A. (2004). VIP (etoposide, ifosfamide, cisplatin) in adult patients with recurrent or refractory Ewing sarcoma family of tumors. Am. J. Clin. Oncol..

[30] He L., Wang X., Luo D. (2011). Synthesis of Curcumin Niconate. China J. Chin. Mater. Med..

[31] Guo J., He Q., Guo Y., Tuo Q., Liao D., Yan J. (2018). Determination of equilibrium solubility and apparent oil-water partition coefficient of nicotinate-curcumin ester. Zhongguo Yao Shi.

[32] Mortezaee K., Salehi E., Mirtavoos-Mahyari H., Motevaseli E., Najafi M., Farhood B., Rosengren R.J., Sahebkar A. (2019). Mechanisms of apoptosis modulation by curcumin: Implications for cancer therapy. J. Cell. Physiol..

[33] Mou S., Zhou Z., He Y., Liu F., Gong L. (2017). Curcumin inhibits cell proliferation and promotes apoptosis of laryngeal cancer cells through Bcl-2 and PI3K/Akt, and by upregulating miR-15a. Oncol. Lett..

[34] Zhu Y., Bu S. (2017). Curcumin Induces Autophagy, Apoptosis, and Cell Cycle Arrest in Human Pancreatic Cancer Cells. Evid. Based Complement. Altern. Med..

[35] El-Deiry W.S. (2016). p21(WAF1) Mediates Cell-Cycle Inhibition, Relevant to Cancer Suppression and Therapy. Cancer Res..

[36] Cao A.L., Tang Q.F., Zhou W.C., Qiu Y.Y., Hu S.J., Yin P.H. (2015). Ras/ERK signaling pathway is involved in curcumin-induced cell cycle arrest and apoptosis in human gastric carcinoma AGS cells. J. Asian Nat. Prod. Res..

[37] Zhao Z., Li C., Xi H., Gao Y., Xu D. (2015). Curcumin induces apoptosis in pancreatic cancer cells through the induction of forkhead box O1 and inhibition of the PI3K/Akt pathway. Mol. Med. Rep..

[38] Catania A., Barrajon-Catalan E., Nicolosi S., Cicirata F., Micol V. (2013). Immunoliposome encapsulation increases cytotoxic activity and selectivity of curcumin and resveratrol against HER2 overexpressing human breast cancer cells. Breast Cancer Res. Treat..

[39] Safavy A., Raisch K.P., Mantena S., Sanford L.L., Sham S.W., Krishna N.R., Bonner J.A. (2007). Design and development of water-soluble curcumin conjugates as potential anticancer agents. J. Med. Chem..

[40] Sun M., Su X., Ding B., He X., Liu X., Yu A., Lou H., Zhai G. (2012). Advances in nanotechnology-based delivery systems for curcumin. Nanomedicine.

[41] Mohanty C., Acharya S., Mohanty A.K., Dilnawaz F., Sahoo S.K. (2010). Curcumin-encapsulated MePEG/PCL diblock copolymeric micelles: A novel controlled delivery vehicle for cancer therapy. Nanomedicine.

[42] Yan R., Zu X., Ma J., Liu Z., Adeyanju M., Cao D. (2007). Aldo-keto reductase family 1 B10 gene silencing results in growth inhibition of colorectal cancer cells: Implication for cancer intervention. Int. J. Cancer J. Int. Du Cancer.

[43] Zu X., Yan R., Robbins S., Krishack P.A., Liao D.F., Cao D. (2007). Reduced 293T cell susceptibility to acrolein due to aldose reductase-like-1 protein expression. Toxicol. Sci..

[44] Bu Y., Li X., He Y., Huang C., Shen Y., Cao Y., Huang D., Cai C., Wang Y., Wang Z. (2016). A phosphomimetic mutant of RelA/p65 at Ser536 induces apoptosis and senescence: An implication for tumor-suppressive role of Ser536 phosphorylation. Int. J. Cancer.

[45] Wang C., Yan R., Luo D., Watabe K., Liao D.F., Cao D. (2009). Aldo-keto reductase family 1 member B10 promotes cell survival by regulating lipid synthesis and eliminating carbonyls. J. Biol. Chem..

