# Supplementary material for: Curcumin Nicotinate Selectively Induces Cancer Cell Apoptosis and Cycle Arrest through a P53-Mediated Mechanism
Source: Molecules. 2019 Nov 18;24(22):4179. doi: 10.3390/molecules24224179 (PMC6891632; doi:10.3390/molecules24224179)
Supplement: Supplementary file 1 [file molecules-24-04179-s001.pdf]

Supplementary Data

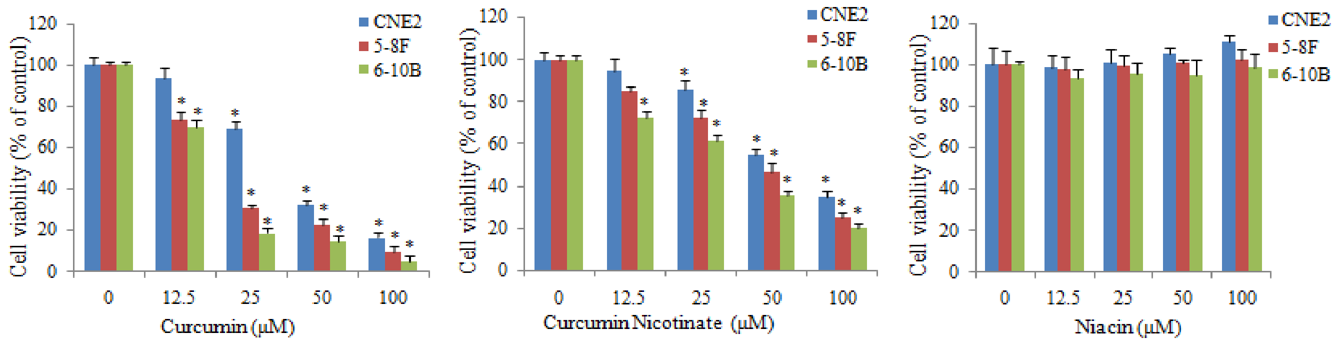

Figure S1. Antiproliferative activity of curcumin nicotinate in nasopharyngeal cancer cells. Nasopharyngeal cancer (NPC) cells CNE2, 5-8F and 6-10B were treated for 72 hours with niacin, curcumin nicotinate or curcumin at indicated concentrations. Cell viability was determined by MTT assays as described in Materials and Methods. Data were analyzed by one-way ANOVA analysis. * $p<0.05$ compared to control cells.

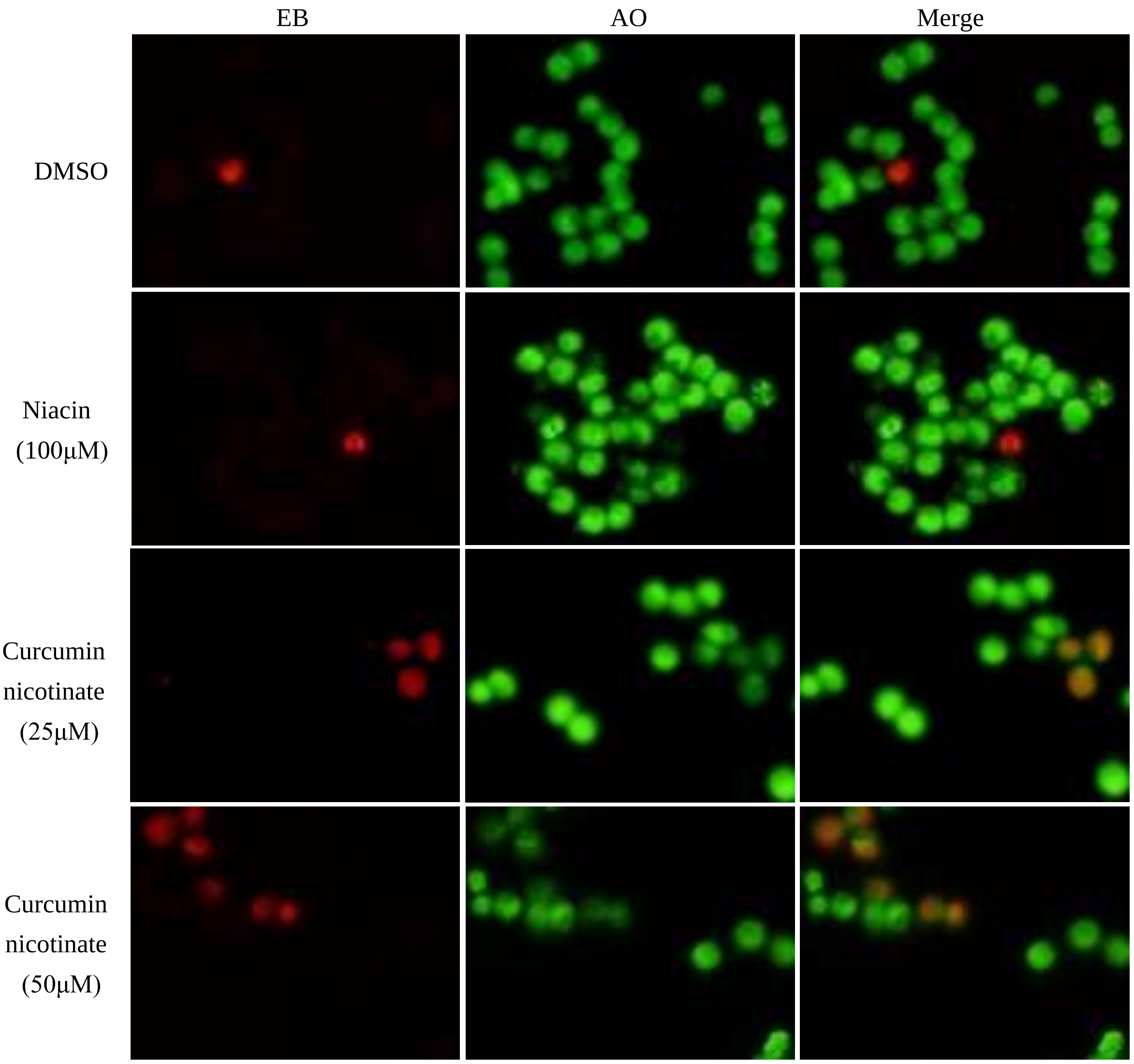

Figure S2. Curcumin nicotinate-induced apoptosis detected by AO/EB staining. HCT116 cells were treated with mock (DMSO), niacin or curcumin nicotinate for 36 hours and then subjected to AO/EB staining as described in Materials and Methods. Red cells indicate apoptotic cells. Magnification, 200x.

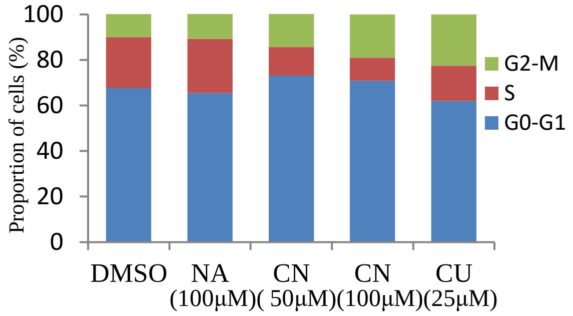

Figure S3. MCF-7 cell cycle arrest induced by curcumin nicotinate. MCF-7 cells were treated for 36 hours with mock (DMSO), niacin, curcumin nicotinate or curcumin and then collected for cell cycle distribution a
